# Exosome Loaded Protein Hydrogel for Enhanced Gelation Kinetics and Wound Healing

**DOI:** 10.1021/acsabm.4c00569

**Published:** 2024-08-22

**Authors:** Dustin Britton, Dianny Almanzar, Yingxin Xiao, Hao-Wei Shih, Jakub Legocki, Piul Rabbani, Jin Kim Montclare

**Affiliations:** † Department of Chemical and Biomolecular Engineering, New York University Tandon School of Engineering, Brooklyn, New York 11201, United States; § Hansjörg Wyss Department of Plastic Surgery, New York University School of Medicine, New York, New York, 10016, United States; ¶ Bernard and Irene Schwartz Center for Biomedical Imaging, Department of Radiology, New York University School of Medicine, New York, New York, 10016, United States; ∇ Department of Chemistry, New York University, New York, New York, 10012, United States; ° Department of Biomaterials, New York University College of Dentistry, New York, New York, 10010, United States; ¥ Department of Biomedical Engineering, New York University, New York, New York 11201, United States

**Keywords:** Protein engineering, hydrogels, exosomes, wound healing, gelation, protein-hybrid, protein-exosome, diabetes

## Abstract

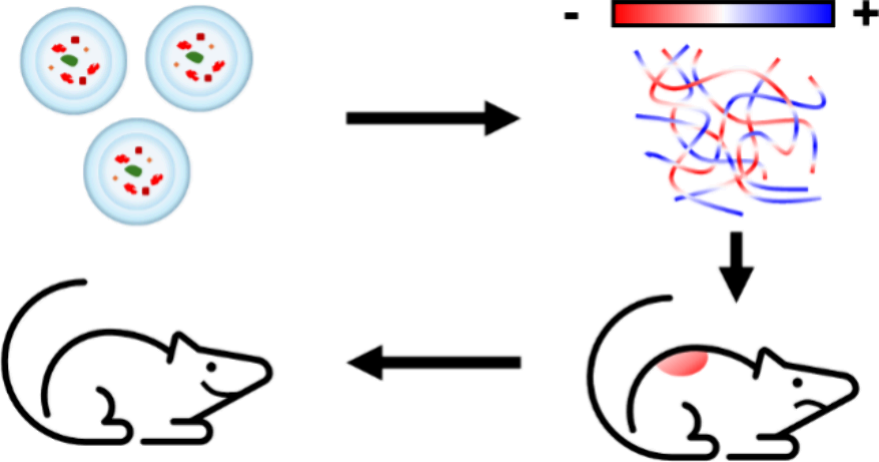

Exosomes are being increasingly explored in biomedical research for wound healing applications. Exosomes can improve blood circulation and endocrine signaling, resulting in enhanced cell regeneration. However, exosome treatments suffer from low retention and bioavailability of exosomes at the wound site. Hydrogels are a popular tool for drug delivery due to their ability to encapsulate drugs in their network and allow for targeted release. Recently, hydrogels have proven to be an effective method to provide increased rates of wound healing when combined with exosomes that can be applied noninvasively. We have designed a series of single-domain protein-based hydrogels capable of physical cross-linking and upper critical solution temperature (UCST) behavior. Hydrogel variant Q5, previously designed with improved UCST behavior and a significantly enhanced gelation rate, is selected as a candidate for encapsulation release of exosomes dubbed Q5Exo. Q5Exo exhibits low critical gelation times and significant decreases in wound healing times in a diabetic mouse wound model showing promise as an exosome-based drug delivery tool and for future hybrid, noninvasive protein-exosome design.

## Introduction

Diabetes is a common disease affecting 13% of American adults^1,2^ where delayed healing is often a problem, and if untreated, may lead to amputation,^3^ necessitating facile and effective methods to treat diabetic wounds. Diabetic wounds are characterized by long-term inflammation, hypoxia, and angiogenesis, which causes this hallmark healing delay.^4^ With particular relevance to wound healing, exosomes—small membranous nanovesicles—have emerged as a promising tool in the field of biomedical applications.^5^ Exosomes contain a list of biological contents including nucleic acids, proteins, and lipids that are able to regulate intercellular communication such as paracrine or endocrine signaling.^6^ Specifically, exosomes derived from mesenchymal stem cells (MSCs), such as adipose-derived stem cells, have shown the ability to promote wound healing in animal models.^7,8^ Exosome-promoted wound healing has been reported to be associated with reducing inflammatory responses via interaction with a variety of immune and tissue cells as well as by promoting pro-angiogenic environments and extracellular matrix deposition through activation of endothelial cells and fibroblasts.^5^ Exosomes offer the distinct advantages of being capable of bypassing concerns associated with stem cell transplantation, such as unwanted growth and immune reactions.^9,10^ Despite being successful in showing their potential to facilitate wound closure in diabetic mouse models, repeated administration subcutaneously or intraveneously may be required.^5^

One such solution to overcome repeated exosome treatment is the combination of exosomes with hydrogels. Hydrogels, made of a network of chemically or physically cross-linked polymers, can be used to encapsulate exosomes in their matrix.^11^ Akin to a wound dressing, this approach is noninvasive and does not require injection by syringe.^12,13^ Hydrogels have been used alone as wound dressings to help in healing and are considered promising due to their high biocompatibility and their ability to hydrate the application site.^14^ Tran et al. have shown that a hydrogel composed of chitosan-conjugated rutin provides a soluble and interactive biomaterial for wounds.^14^ Hydrogels can also be used to encapsulate exosomes inside their network for increased biotherapeutic efficacy, particularly for diabetic wounds.^11^ For an extensive review of these hydrogels, Li and Wu^4^ and Safari et al.^11^ have listed various hydrogel/exosome combinations and their mechanisms. In these systems, the fastest wound closure rates are generated by hydrogel-exosome combinations, followed second by exosomes alone, and third by hydrogels alone, when compared to the diabetic control.^15−17^ In all reported hydrogel-exosome systems, the hydrogel is not protein-based.^4,11^

We have previously developed a single coiled-coil domain protein-based hydrogel, dubbed Q, that is capable of undergoing a solution-to-gel transition at low temperatures known as upper critical solution temperature (UCST) gelation behavior.^18^ Q undergoes supramolecular assembly into nanofibers that physically cross-link at high concentrations.^18^ Since then, we have established criteria to design coiled-coil hydrogels toward increased UCSTs, increased material strengths, and faster gelation times using Rosetta Score and Poisson–Boltzmann calculated electrostatic potential energies.^19,20^ Based on the importance of mechanically strong hydrogels for enrichment of the wound bed,^21^ protein-based hydrogels, possessing modular sequences using an interchangeable amino acid library, may offer a platform for vast improvement in wound closure rates.

To demonstrate the application of designed protein-based coiled-coil hydrogels, we use recent variant, Q5, designed by automating the selection of mutations for improved thermostability using a Rosetta score-based Monte Carlo search.^19^ Exosomes encapsulated by Q5 are utilized as a candidate hydrogel-exosome system for diabetic wound healing by facile application as a noninjectable, topical wound dressing compared to standard applications of exosomes by subcutaneous or intravenous injection.^5^ In using our hydrogel-exosome material, Q5Exo, we demonstrate the ability to use protein-based systems for encapsulation of exosomes and substantially decrease the time for wound healing in diabetic mice by topical application, showing promise for future protein-based hydrogels in wound healing applications.

## Materials and Methods

### Materials

Chemically competent M15MA *E. coli* cells were gifted from David Tirrell at California Institute of Technology. Bacto-tryptone, sodium chloride (NaCl), yeast extract, tryptic soy agar (TSA), ampicillin sodium salt, sodium phosphate dibasic anhydrous (Na_2_HPO_4_), sodium hydroxide (NaOH), urea, dextrose monohydrate (d-glucose), magnesium sulfate (MgSO_4_), calcium chloride (CaCl_2_), manganese chloride tetrahydrate (MnCl_2_·4H_2_O), cobaltous chloride hexahydrate (CoCl_2_·6H_2_O), isopropyl β-d-1-thiogalactopyranoside (IPTG), Pierce bicinchoninic acid (BCA) assay kit, Pierce snakeskin dialysis tubing 3.5 K molecular weight cutoff (MWCO), sodium dodecyl sulfate (SDS), Nunc MicroWell 96-Well plates, and BD Clay Adams glass microscopy slides were acquired from Thermo Fisher Scientific. The 20 naturally occurring amino acids, dimethyl sulfoxide (DMSO), nickel(III) chloride hexahydrate (NiCl_2_·6H_2_O), sodium molybdate dihydrate (Na_2_MoO_4_·2H_2_O), iron(III) chloride (FeCl_3_), iron(II) chloride tetrahydrate (FeCl_2_·4H_2_O), thiamine hydrochloride (vitamin B), curcumin, and copper(II) sulfate pentahydrate (CuSO_4_·5H_2_O) were purchased from Sigma–Aldrich. Hydrochloric acid (HCl) and Coomassie Brilliant Blue G-250 were purchased from VWR. HiTrap FF 5 mL columns for protein purification were purchased from Cytiva Life Sciences. Macrosep and Microsep Advance Centrifugal Devices 3K MWCO and 0.2 μm syringe filters were purchased from PALL. Acrylamide/bis solution (30%) 29:1, and natural polypeptide sodium dodecyl sulfate–polyacrylamide gel electrophoresis (SDS-PAGE) standard were purchased from Bio-Rad. Copper(II) chloride anhydrous (CuCl_2_), sodium selenite (Na_2_SeO_3_), and imidazole were purchased from Acros Organics. Formvar/carbon-coated copper grids (FCF400-Cu) and 1% uranyl acetate for transmission electron microscopy were purchased from Electron Microscopy Sciences.

### Expression and Purification of Q5

The expression of Q5 proteins were performed using previously described methods.^19^ The pQE60/Q5 plasmid was ordered from Genscript. Methionine-auxotrophic M15MA *E. coli* cells, a gift from David Tirell,^22^ were used for the expression of Q5. Q5 was transformed and plated onto tryptic soy agar (TSA) plates and incubated at 37 °C overnight. Colonies were selected and inoculated in M9 minimal media (0.5 M Na_2_HPO_4_, 0.22 M KH_2_PO_4_, 0.08 M NaCl, 0.18 M NH_4_Cl, d-glucose (100 μg/mL), magnesium sulfate (1 mM), and calcium chloride (0.1 mM)) containing all 20 natural amino acids (100 μg/mL) and supplemented with ampicillin (200 μg/mL), kanamyacin (35 μg/mL), and vitamin B (35 μg/mL) and incubated overnight at 37 °C and shaken at 350 rpm. Starter cultures were transferred to 400 mL of supplemented M9 minimal media and allowed to grow until an optical density of 600 nm (OD_600_) reached ∼0.8, where protein expression was induced by adding 200 μg/mL of IPTG. The cells were then incubated at 37 °C and 350 rpm for 3 h. Subsequently, cell harvest was centrifuged at 5000*g*, 4 °C, for 30 min using an Avanti J-25 centrifuge (Beckman Coulter). The harvested cells were stored at −20 °C until purification. Expression of Q5 protein was confirmed by performing 12% SDS-PAGE analysis. For purification Q5 was subjected to affinity chromatography on a cobalt-charged HiTrap IMAC FF 5 mL column, using Buffer A (50 mM Tris-HCl, 500 mM NaCl, pH 8.0). Elution of the protein was performed by applying a gradient of Buffer B (50 mM Tris-HCl, 500 mM NaCl, 500 mM imidazole, pH 8.0), with the imidazole concentration ranging from 5 mM to 500 mM. Pure fractions were dialyzed in six consecutive 5 L volumes of Buffer A and concentrated to 2 mM using 3 kDa Macrosep centrifugal filters (Pall). Protein purity was confirmed by 12% SDS-PAGE (Figure S1), and their concentrations were determined by the BCA assay.

### Microrheology

The protein concentration was adjusted to 2 mM, determined by the BCA assay, and immediately divided into 27.7 μL aliquots in 200 μL PCR tubes. Next, 1% v/v (or 0.3 μL) of 1-μm-diameter red polystyrene fluorometric beads (FluoSpheres) were added to each sample. The sample was then loaded into a glass capillary tube using capillary action and sealed to prevent evaporation. The capillary tube was affixed to a microscopy slide using a fast-curing epoxy adhesive (JB Weld).^23^ Microrheology methods and calculations were consistent with previously described protocols for protein hydrogels.^23,24^ Briefly, the samples were imaged every 12 h thereafter, using an inverted fluorescent microscope (ZEISS Microscopy) at 40× magnification with 2 × 2 binning. To avoid sedimentation of the fluorometric beads, the slides were incubated on a rotisserie at 8 rpm between imaging sessions. Each image series consisted of 300 frames with a lag time (τ) of 0.037 s between each frame. Multiple particle tracking (MPT) data was utilized to determine the completion of hydrogel equilibration and cessation of measurements (in this case, at 60 h)^23^ using MATLAB (MathWorks, R201b) algorithms developed by Kilfoil and co-workers,^25^ Dufresne et al.,^26^ Blair and co-workers,^27^ and O’Neill et al.^28^ Images were first stacked and converted to grayscale. Then, superposition analysis was used, where all curves of the mean-square displacement were mathematically superimposed onto the 0 h curves. Upon divergence of curve superimposition to 0 h, curves were then superimposed to the final time point curve. The time at which data diverged indicated the window for the sol–gel transition. The intersection of the master sol-state and master gel-state curves represent the critical gelation time (*t*_gel_), corresponding to the critical relaxation exponent (*n*_c_).

### Cell Culture

An immortalized adipose-derived mesenchymal stromal cell (ADSC) line was used, generated by transduction with human telomerase lentivirus Htert (ATCC, SCRC-4000, Manassas, VA, USA). One million cells were plated into three 150 mm diameter plates, in 18 mL of low glucose MEM-alpha with 15% FBS (ThermoFisher Scientific), 1% penicillin and streptomycin (Sigma–Aldrich), and 1% MEM nonessential amino acid solution (Sigma–Aldrich), and incubated at 37 °C, 5% CO_2_. When the plates were 80% confluent, plates were passaged using 3× dilutions until passage 3. Cells were detached by using 6 mL of 0.25% Trypsin-EDTA (Invitrogen) and neutralized with equal amounts of complete media.

### Exosome Prep Isolation

When cells reached 70% confluency, complete media in the plates were replaced with media supplemented with exosome-free FBS (ThermoFisher Scientific). We adapted an established protocol of exosome isolation.^29^ The conditioned media was collected 48 h later in 50 mL centrifuge tubes. The tubes were centrifuged for 20 min at 2000*g*, 4 °C. The supernatant was transferred into polycarbonate 70 mL tubes (Beckman Coulter, No. 355622), leaving behind the pellet containing dead cells. Each was weighed to ensure an equal weight. The tubes were centrifuged for 30 min at 10 000*g*, 4 °C in a F37L-8x100 rotor (Thermo Scientific) and a wX+ Ultra Series centrifuge (ThermoScientific, No. 75000100). All tubes were weighed using PBS with Ca^2+^/Mg^2+^ as necessary, to give equal final weights. The supernatant was collected and filtered through a 0.22 μm filter (ThermoScientific, No. 126-0020). The filtered supernatant was transferred to new 70 mL polycarbonate ultracentrifuge tubes and centrifuged for 90 min at 100 000*g*, 4 °C in a F37L-8x100 rotor. The pellet containing the small extracellular vesicles (exosome) prep in 1 mL was resuspended with remaining supernatant and then transferred to 10.4 mL polycarbonate tubes (Beckman Coulter, Inc.). The tubes were centrifuged for 90 min at 100 000*g*, 4 °C in a 70.1 Ti Rotor with a Sorvall WX Ultra Series centrifuge (ThermoScientific, No. 46901). The pellets containing the exosome prep in 200–400 μL of residual supernatant were resuspended and aliquoted before the evaluation of size and concentration by NTA using Zetaview (ParticleMetrix, PMX-420 QUATT).

### Exosome Prep Labeling with Fluorescence

Exosome preps were fluorescently labeled using ExoGlow In-Vivo Labeling Kit (EXOGV900A-1, SBI Biosciences), which uses a proprietary nonlipophilic dye that emits in the near-infrared (NIR) range with excitation at 785 nm and emission at 806 nm. The manufacturer’s protocol was followed. Briefly, the dye stock solution was prepared with 25 μL of anhydrous DMSO (Fisher Scientific). Approximately 2 μL of the stock dye solution was added to the exosome preparation in 500 μL of PBS and incubated for 45 min at room temperature. The labeled exosome prep was precipitated by adding 167 μL of ExoQuick-TC and incubating the tube overnight at 4 °C. The mixture was spun at 13 000*g* for 10 min to recover the exosomes in the pellet. The pellet was resuspended in 160 μL of PBS and used immediately for encapsulation into hydrogels.

### Exosome Loading

Q5Exo samples were prepared by mixing concentrated exosomes into freshly concentrated Q5 protein samples. Exosomes dissolved in PBS were thawed at 1 × 10^8^/μL exosomes and 30 μL were added to 120 μL Q5 after concentration to 2.5 mM, as confirmed by BCA analysis. Samples were thoroughly mixed by pipetting before incubation at 4 °C until complete transition into a hydrogel was confirmed by sigmoidal fit analysis of microrheologically assessed multiple particle tracking (MPT) (Figure S2).

### Circular Dichroism Spectroscopy

To evaluate the secondary structure of the samples, we employed circular dichroism (CD) using a spectrometer (Jasco, Model J-815) equipped with a PTC-423S single position Peltier temperature control system. Wavelength scans were conducted in the range of 190 to 250 nm, with 1 nm step sizes, using 15 μM samples at a temperature of 25 °C. The mean residue ellipticity (MRE) was determined using established methods.^30^ The MRE values at 222 and 208 nm, as well as their ratios, were utilized to assess the relative helical content.

### Attenuated Total Reflectance–Fourier Transform Infrared Spectroscopy

To evaluate the secondary structure of Q5Exo samples under representative buffer and concentration conditions, ATR-FTIR spectroscopy was employed. Following gelation of Q5Exo hydrogels, 5 μL of Q5Exo hydrogels were loaded onto a Nicolet 6700 Fourier transform infrared spectrometer equipped with a mercury cadmium telluride (MCT)-A detector and a diamond crystal for 1 min. Spectra was collected from 4000 to 400 cm^–1^ with a 4.0 cm^–1^ resolution. Spectra was analyzed by buffer-subtraction and deconvolution of spectra from 1700 to 1600 cm^–1^. Spectra was deconvoluted using Gaussian functions in PeakFit software until *R*^2^ values were >0.99.

### Rheology

To evaluate the mechanical stability of the Q5 and Q5Exo hydrogels, a stress-controlled rheometer (Discovery Hybrid Rheometer 2, TA Instruments) with a parallel plate geometry was utilized. Once the 2 mM sample had completely gelled at 4 °C, it was placed between the lower and upper plates with an 8 mm diameter and a 0.2 mm gap. The strain and frequency settings were determined based on previous studies on Q hydrogels.^18,20^ The storage modulus (*G*′) and loss modulus (*G*″) were measured across a frequency range of 0.1–10 Hz, employing a 5% oscillation strain.

### Transmission Electron Microscopy

Transmission electron microscopy (TEM) images were acquired by using a FEI Talos L120C transmission electron microscopy (TEM) system. For the visualization of protein fibers, samples were diluted to a concentration of 50 μM. Subsequently, 3 μL of the diluted samples was carefully spotted onto Formvar/carbon-coated copper grids. A washing step with 5 μL of water was performed, followed by staining with a 1% (v/v) solution of uranyl acetate, with each step involving a 1 min incubation at room temperature. The sizing of the fibrils was measured using ImageJ software (Version 1.52q).^31^

### Exosome Release from Q5Exo

Biomolecule release profiles from protein-based hydrogels were adapted from previously established protocols.^24,32^ Following preparation of 150 μL samples of 2 mM Q5Exo hydrogels loaded with 3e^9^ exosomes, 300 μL of 50 mM TrisHCl and 500 mM NaCl (pH 8.0) buffer was used to incubate with the samples while shaking at 300 rpm and 37 °C (Thermomixer R, Eppendorf). Periodically, samples were removed and gently centrifuged at 2500 rpm for 2 min. The supernatant was collected and spectrophotometrically assessed for NIR-labeled exosomes using a Duetta fluorescence (785/836 ex/em) and absorbance spectrometer (Horiba Scientific) and for protein concentration using a BCA assay. The experiment was concluded when signal differences for NIR or protein concentration in the BCA assay were no longer detected.

### Wound Model

All animal protocols were approved by the New York University School of Medicine Institutional Animal Care and Use Committee. Type 2 diabetic (Lepr^db/db^) mice were obtained from Jackson Laboratory (Bar Harbor, ME, USA). The mice were anesthetized using 2% isoflurane, and the foot pad pinch test was used to confirm that the mice were completely sedated. The hair was shaved from the mouse dorsum and Nair hair removal cream was used to remove remaining hair shafts. Two 10-mm-diameter full-thickness wounds were created, including the panniculus carnosus, using a punch biopsy tool. To prevent the panniculus carnosus from contracting the wound and resulting in premature closure, the wound was splinted with a 0.6-mm-thick silicone stent with an inner diameter of 10 mm and an outer diameter of 20 mm (using silicone sheets from Grace Bio-Laboratories, Bend, OR, USA). Silk 4–0 braided reverse cutting suture (Henry Schein, Inc., Melville, NY, USA) was used to secure the stent to the skin surrounding the wound. To minimize scratching, chewing, and biting of the sutures by the mice, an occlusive adhesive dressing with a 12 mm window overlying the silicone stent was applied. The window allows air to exchange to the wound, while covering the actual sutures. Buprenorphine was administered for analgesia for 3 days post-operatively. Approximately 150 μL of 2 mM Q5 hydrogel with encapsulated exosomes (Q5Exo) was applied (Figure S3), containing 3 × 10^9^ exosomes per wound or equal exosome numbers, or PBS was injected once circumferentially. The blood glucose was measured once per week. Q5Exo dressings were not changed during the treatment of diabetic wounds. The wounds were photographed at regular intervals to capture the wound closure over time. Using ImageJ, the photographic results were quantified by measuring the area of the scab relative to the internal diameter of the 10 mm silicone stent of the same wound, identical to the original wound diameter. Excisional or open wound area was calculated as (unhealed wound area)/(original wound area) × 100 (expressed as a percentage). The wound closure was assessed by the similarity of the re-epithelialized skin to unwounded intact skin, with regard to color, upon gross visual inspection and appearance.

### In Vivo Imaging of Mouse Wounds

The in vivo imaging system (IVIS) (PerkinElmer IVIS Lumina III) was employed using Living Image software (PerkinElmer) to capture the 785/836 ex/em of NIR (exosome prep label) and 410/470 ex/em for autofluorescence (AF) from the Q5 fibers. A spectral scan was performed to best capture the desired fluorescent wavelengths with the available detection capacity of the IVIS. For NIR, the closest match was 780/845 ex/em, and for AF the closest was 420/520 ex/em. All data were processed using Aura software, version 4.0 (AZ, USA).

### Statistical Analysis

Our data of three biological replicates was represented as mean ± standard deviation. Prism 10 (GraphPad software, MA, USA) was used to perform a one-way ANOVA followed by Tukey’s posthoc test for multiple comparisons. Prism was also employed for statistical analysis using a Student’s *t*-test.

## Results and Discussion

### Structure and Nanoassembly

**Figure 1:**
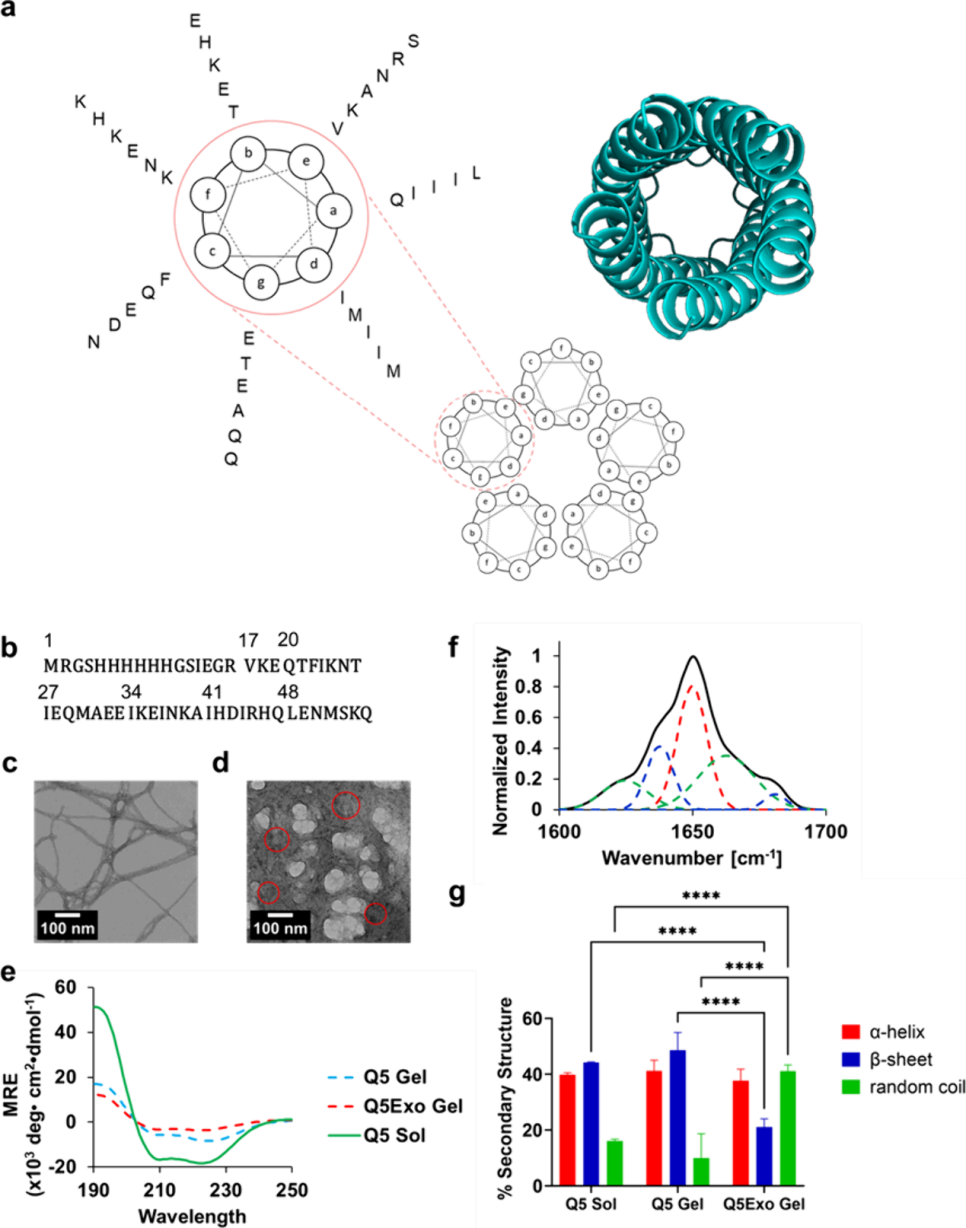
(a) Helical wheel diagram of the pentameric coiled-coil with a cartoon ribbon diagram of Q5 for reference. One helical wheel of Q5 is highlighted with corresponding helical wheel positions matching the location of residues in order (inside to outside) beginning with a partial heptad (VKE) starting at the *e* helical wheel position. (b) Sequence of Q5 with corresponding sequence numbers indicated at the top left of the start of the histag, partial heptad (VKE), and full heptads. (c) TEM image of Q5 and (d) Q5Exo indicates increasing physical cross-linking upon addition of exosomes to Q5. Red circles outline round morphology of candidate exosome sites. (e) Average wavelength scans in circular dichroism (CD) spectroscopy of Q5 before incubation at 4 °C (Sol) and after incubation at 4 °C with (Gel + Exo) and without (Gel) exosomes. Spectra represent the average of three independent trials. [Data for circular dichroism measurements of Q5 Gel and Q5 Sol is adapted from ref (19), licensed under CC BY 4.0.] (f) Representative FTIR spectra of the Q5Exo hydrogel. Spectra is deconvoluted for α-helical (red), β-sheet (blue), and random coil (green) secondary structure. (g) Average percentage of deconvoluted secondary structure from FTIR spectra for Q5 in the solution-state prior to incubation at 4 °C (Q5 Sol) and in the gel-state after incubation at 4 °C (Q5 Gel) and of Q5Exo in the gel-state (Q5Exo Gel). Error bars represent the standard deviations of three independent trials.

Q5 was designed using a probabilistic Rosetta-based Monte Carlo search to generate a variant with an increased upper critical solution temperature (UCST) by automating selection of residues that were most likely capable of providing increased stability measured by the Rosetta score at a given position. Charged or neutral residues were used as selection criteria for residues in the “b”, “c”, and “f” helical wheel positions, whereas polar or neutral residues used as criteria for the “a” and “d” helical wheel positions of the coiled-coil (Figure 1a). Coiled-coil sequences follow a heptad-repeating system denoted by positions in the “a”, “b”, “c”, “d”, “e”, “f”, and “g” helical wheel where self-assembly is dictated primarily by hydrophobic interaction in the “a” and “d” helical wheel position and supramolecular assembly is primarily dictated by charged interactions in the “b”, “c”, and “f” helical wheel positions.^33^ The final sequence of Q5 (Figure 1b) possessed a decreased electrostatic potential difference of surface-facing residues–in the “b”, “c”, and “f” helical wheel positions–between the N- and C-terminus. We previously established that a lower electrostatic potential difference, known as ΔEE_bcf_, to be well correlated to decreased fiber diameters of fiber-forming^34^ and gel-forming systems.^19,32^ Indeed, Q5 exhibited the lowest average fiber diameters of our coiled-coil hydrogels.^19^ The decreased fiber diameters further allowed for increased physical cross-linking, increased mechanical strength, and increase rate of gelation.^19^

To investigate the impact of exosome encapsulation by Q5 on its nanoassembly, transmission electron microscopy (TEM) was employed. Q5 exhibited physical cross-linking typical of previous hydrogels and possessed average fiber diameters of 22.2 ± 8.4 nm (Figure 1c).^19^ When incubated at 4 °C with exosomes, the resulting Q5Exo revealed a substantial increase in physical crosslinks and fiber–fiber interactions (Figure 1d), suggesting an increased propensity for gel-like nanostructures, shown by densely stained proteins. However, average fiber diameters remained relatively similar at 31.2 ± 5.6 nm, statistically insignificant by an unpaired *t*-test. With exosomes acting as interstitial sites for protein cross-linking, distinguishing exosomes and aggregate proteins is difficult. However, the appearance of circular cuplike morphologies with ∼100 nm diameters in stained TEM regions^35^ suggest the sites of exosomes (Figure 1d).

The structural impact of exosomes on Q5 was assessed by circular dichroism (CD) spectroscopy (Figure 1e). Q5 previously exhibited signals expected of coiled-coil hydrogels possessing a strong double minima at 208 and 222 nm of −15 600 ± 1800 deg cm^2^ dmol^–1^ and −18 300 ± 1000 deg cm^2^ dmol^–1^, respectively, as a solution prior to incubation at 4 °C followed by a significant dampening to −4900 ± 1400 deg cm^2^ dmol^–1^ and −7700 ± 1700 deg cm^2^ dmol^–1^ at 208 and 222 nm, respectively, upon gelation after incubation at 4 °C.^19^ Signal dampening was previously associated with phase transition into a hydrogel.^19^ Following encapsulation of exosomes and incubation at 4 °C, Q5Exo transitioned into a hydrogel with a double minima at 208 and 222 nm of −3500 ± 1000 deg cm^2^ dmol^–1^ and −4400 ± 1400 deg cm^2^ dmol^–1^, respectively, an even greater dampening of the CD signal. Q5 and Q5Exo both demonstrated strong coiled-coil structure possessing 222/208 ratios of 1.2 ± 0.1 as a solution, 1.6 ± 0.1 as a gel, and 1.3 ± 0.2 as a gel bound with exosomes where ratios of >1 are indicative of helices found together, such as coiled-coils, rather than in isolation.^36−38^ To evaluate changes in the secondary structure at representative buffer conditions and concentrations, ATR-FTIR spectroscopy was employed for Q5Exo gels. Deconvoluted spectra of Q5Exo demonstrated a strong portion of helical secondary structure possessing 37.7% ± 4.1%, consistent with previous Q hydrogels and minimal changes compared to solution-state Q5 and gel-state Q5,^19^ before and after incubation of exosomes (see Figures 1f and 1g). Q5Exo further demonstrated a loss in β-sheet structure at the expense of random coil structure with contents of 21.1% ± 2.9% and 41.1% ± 2.2%, respectively (Figures 1f and 1g). Overall, this loss in structured content indicates that the addition of exosomes may perturb the ability of the protein to form structured content and is consistent with a loss of helical content presented in CD measurements.

### Rheology

**Figure 2:**
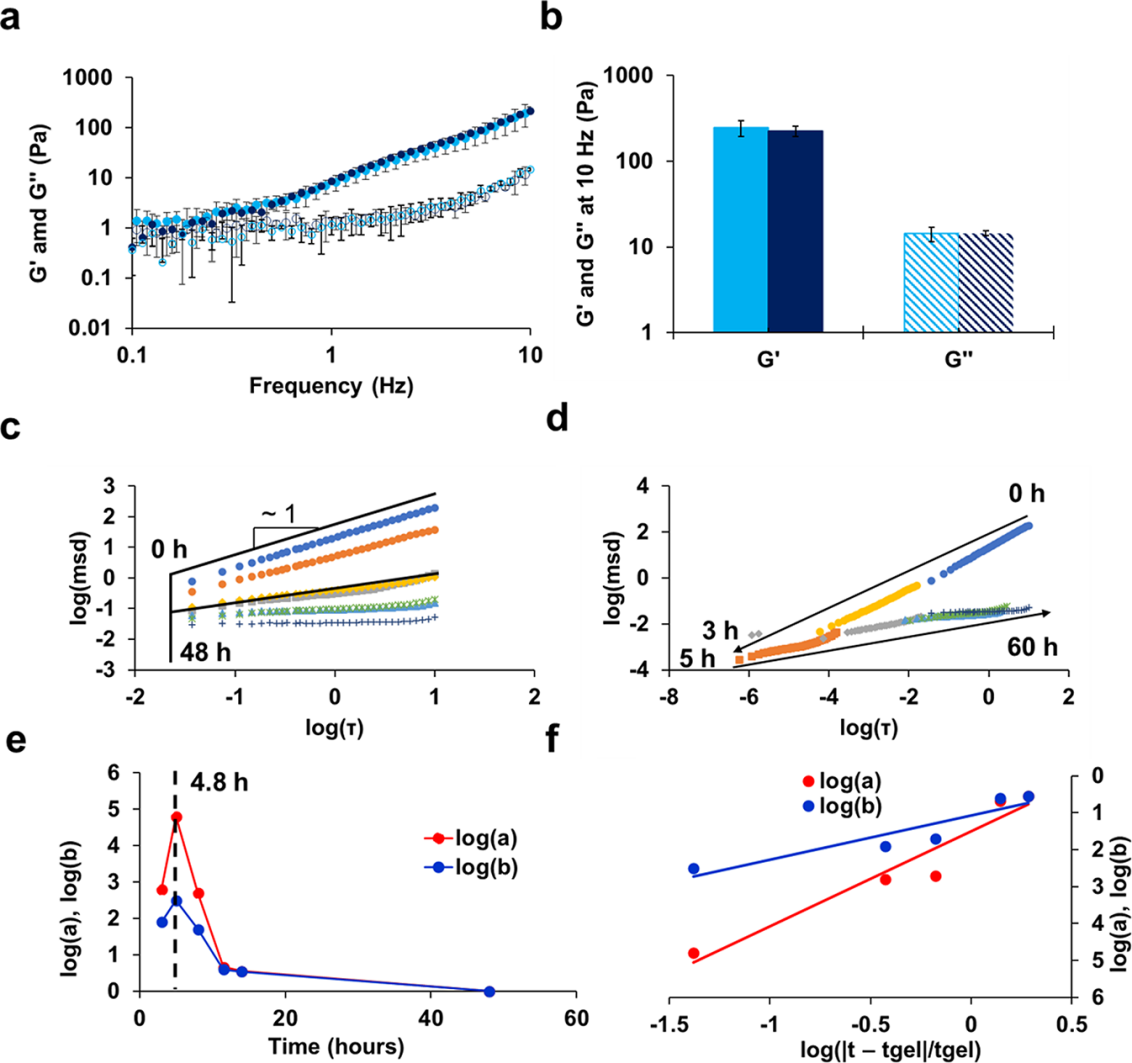
(a) Storage modulus (*G*′, filled circles) and loss modulus (*G*′′, open circles) of Q5 with (dark blue) and without exosomes (light blue) as a function of frequency between 0.1 and 10 Hz after incubation at 4 °C. (b) *G*′ and *G*″ of Q5 (light blue) and Q5Exo (dark blue) after incubation at 4 °C showing a statistically insignificant difference between them. Error bars represent the standard deviation of three independent trials. [Rheology data of Q5 is reproduced for comparison from ref (19), licensed under CC BY 4.0.] (c) Log–log plot of MSD and lag time (τ) for a representative independent trial of Q5Exo determined by MPT using measurement intervals between 0 h (dark blue), 3 h (orange), 5 h (gray), 8 h (yellow), 11.5 h (light blue), 14 h (green), and 48 h (dark gray). (d) Time-cure superposition of MSD vs τ. (e) Logarithmic shift factors for the vertical (log(*a*) shown in red) and horizontal (log(*b*) shown in blue) directions used in the time-cure superposition to determine the value of *t*_gel_. (f) Log–log plot of the shift factors and their distance from *t*_gel_, as determined by the ratio of the logarithmic slopes of the horizontal to vertical shift factor. Panels (c)–(f) are representative of microrheology experiments and corresponding analysis. Plots of the remaining two trials are shown in Figures S4 and S5.

Q5Exo hydrogel material strength and gelation kinetics were assessed using parallel plate rheometry and a high-throughput microrheological assay,^23^ respectively. Storage (*G*′) and loss (*G*′′) moduli were measured using a frequency sweep as done previously.^20^ Negligible differences were revealed between the Q5 and Q5Exo hydrogels at frequencies between 0.1 and 10.0 Hz (Figure 2a). Using 10 Hz, Q5Exo possessed a *G*′ and *G*′′ of 225 ± 32 Pa and 14 ± 1 Pa (Figure 2b), respectively, indicative of gel-like behavior and consistent with Q5 hydrogels without exosomes demonstrated previously.^19^

Rate of Q5Exo gelation was assessed by microrheology after incubation at 4 °C. Multiple particle tracking (MPT) was used to track fluorescent tracer beads over time until negligible mean-square displacement (MSD) changes were observed (Figure S2) and repeated for three independent trials (Figures 2c–f, as well as Figures S4a–S4d and S5a–S5d). Upon binding of exosomes, a solution–gel transition was found to be significantly faster than that without exosomes (Figures 2c–f). Q5Exo possessed a critical time to gelation (*t*_gel_) of just 5.4 ± 1.2 h, compared to 11.5 ± 1.5 h for Q5 alone, *a* > 2-fold improvement. Q5Exo also possessed a critical relaxation exponent of 0.51 ± 0.04, consistent with our hydrogel systems.^19,20,24,32^ Moreover, Q5Exo exhibited a plateau of the logarithmic slope of the particle MSD at the end of its gelation transition of 0.11 ± 0.03, significantly lower than Q5 alone,^19^ where a plateau finalized at 0.39 ± 0.14. This difference suggested a more physically cross-linked behavior from Q5Exo, compared to Q5 at the end of the gel transition, consistent with the comparison of TEM images of Q5 (Figure 1a) and Q5Exo (Figure 1b). Overall, while encapsulation of exosomes did not impact the material strength of Q5Exo, it strongly increased the rate of gelation of the Q5 hydrogel system and generated a more densely cross-linked system.

### Wound Healing

**Figure 3:**
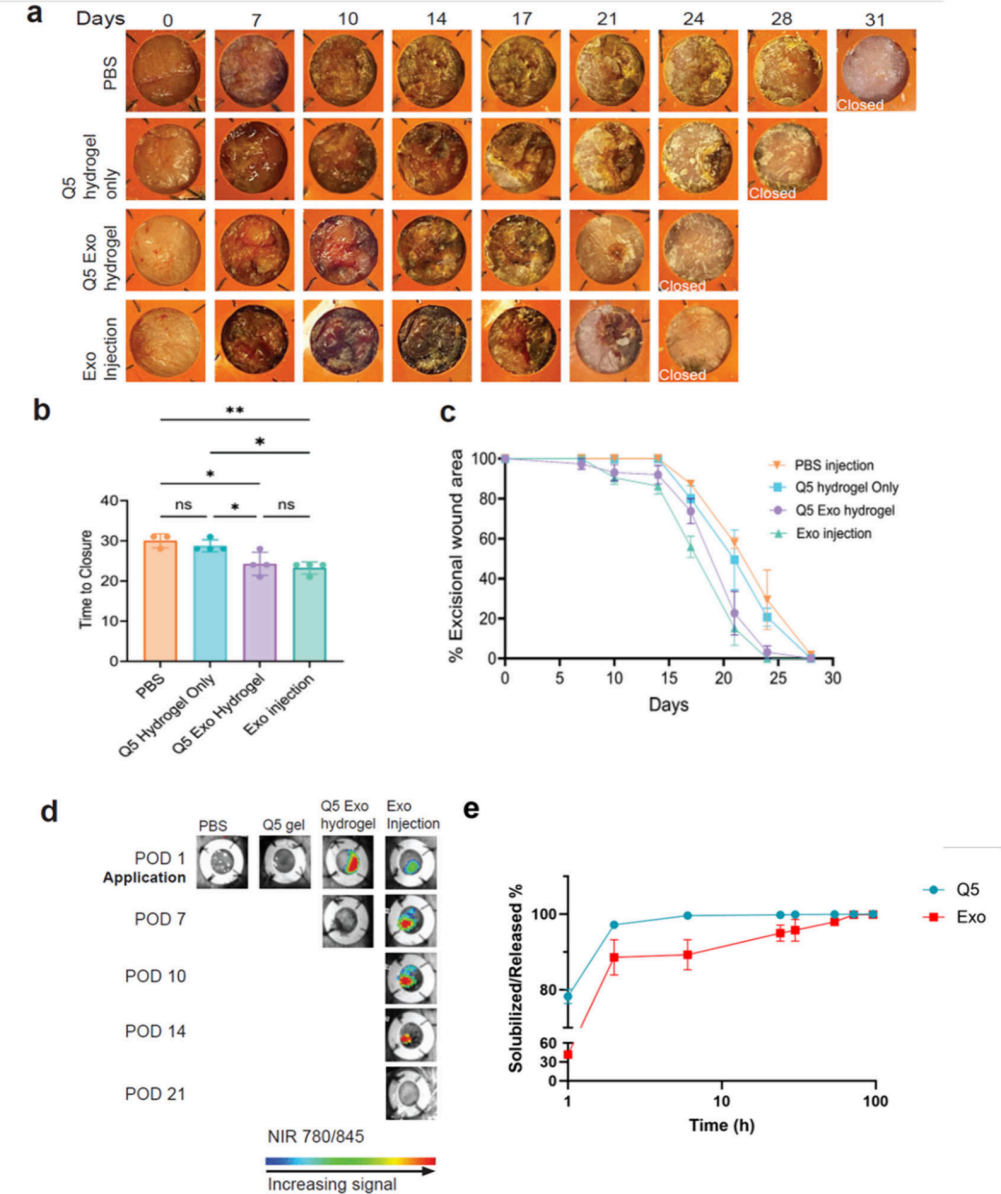
Q5Exo hydrogel affects wound closure in diabetic mice. (a) Photographs of diabetic wounds over time with treatments as indicated. (b) Quantification of time to wound closure, *n* ≥ 3. Error bars represent standard deviation. (Legend: (*) *p* < 0.05, (**) *p* < 0.01.) (c) Percent of unhealed wound area over time, post-excision, and treatments as indicated. Error bars represent standard deviation. (d) In vivo monitoring of treated diabetic wounds. Exosomes are labeled with NIR dye. (e) Sustained release profile of Q5 and NIR-labeled exosomes in Q5Exo in vitro sustained release experiment using BCA assay and 785/836 ex/em by fluorometric spectroscopy, respectively.

To study the effectiveness of exosome-loaded Q5 hydrogel (Q5Exo) on wound closure as a topical treatment, we created stented wounds on adult Lepr^db/db^ diabetic mice, which is an established preclinical model^39,40^ for delayed healing and response to treatments. The stent allows mouse full-thickness wounds to mimic human cutaneous wound healing by allowing the formation of granulation tissue without the contraction of the panniculus carnosus muscle layer. Our photographic analysis demonstrated closure of Q5Exo hydrogel-treated mouse diabetic wounds by 24.3 ± 2.9 days (Figure 3a and 3b). The time to closure is a significant reduction from that of control PBS-treated wounds that closed by 30.0 ± 1.7 days and Q5-only hydrogel-treated wounds that closed by 28.8 ± 1.5 days. Time to closure for exosome injection-treated wounds was on par with that of Q5Exo-treated wounds, with closure by 23.2 ± 1.5 days (Figures 3 b and 3c).

To study the distribution of Q5Exo in mouse wounds in vivo, we used fluorescently labeled exosome preparations to formulate Q5Exo hydrogels. Exosomes were fluorescently labeled with a proprietary nonlipophilic dye (SBI Biosciences) with NIR fluorescence. We chose near-infrared (NIR) to contrast autofluorescence of the Q5 fibers demonstrated recently.^19^ We set out to utilize the autofluorescence of Q5 to trace the hydrogel as a drug delivery vehicle distinctly from the NIR-labeled exosomes. NIR fluorescence and Q5 hydrogel autofluorescence were monitored until a loss of signal. Following hydrogel application at post-operative day 1 (POD1) and in vivo detection of the NIR-labeled exosome prep in the mouse wounds, NIR signal was lost by POD7 (Figure 3d). While we detected the Q5 hydrogel autofluorescence preadministration, we did not detect signal in the mouse wounds (data not shown), indicating Q5 hydrogel autofluorescence also does not provide its own traceability by POD7. NIR-labeled exosome prep injection had the longest detection time in mouse wounds until POD17. Whether the signal is due to the exosome preparation or autofluorescence from a scab is indistinguishable. We did not detect any NIR signal in the Q5-only hydrogel and PBS injection-administered wounds. The relative loss of the NIR signal from exosomes in the Q5Exo-treated group compared to the exosome-injected group can be explained by differences in their administration. Injection of the exosomes may cause a foreign body response and fibrous capsule around the dense exosome formulation, resulting in retention of the NIR signal. In comparison, the topically applied Q5Exo treatment may result in improved distribution and uptake of exosomes, resulting in the NIR signal from the exosomes spreading out, which would correspond with a loss of detectable signal. Blood glucose monitoring demonstrated that none of the local and topical administrative routes that we used in this study affected the hyperglycemic (≥350 mg/dL) status of the mice (Figure S6). Our results strongly suggested that the hydrogel can make exosomes available for skin wound tissues to accelerate diabetic wound closure.

To assess sustained release of exosomes and protein hydrogel erosion, Q5Exo hydrogel samples were incubated in the presence of excess buffer where protein and biomolecule signal (NIR dye of labeled exosomes) were measured over time at 37 °C (Figure 3e).^24,32^ Notably Q5, protein was solubilized immediately upon incubation at 37 °C as seen with previous Q proteins.^24,32^ In contrast, the Q5Exo hydrogel structure appeared to be maintained upon initial incubation. Following 1 h of incubation, Q5Exo hydrogels appeared mostly solubilized with an average solubilization of 78% ± 2%. The protein then exhibited exponential depreciations in solubilization, corresponding to matrix degradation of the protein hydrogel until 72 h. In contrast, release of the exosomes followed an initial burst release to 42% ± 1% at 1 h and 89% ± 5% at 2 h, followed by a subsequent linear release rate of 4% per day (*R*^2^ = 0.91) until 72 h.

Previously, the release of curcumin encapsulated in Q hydrogels also demonstrated a two-phase behavior with an initial burst release followed by a slower rate of release in a second phase. While the time scale of exosome release in Q5 was shorter, both demonstrated a two-phase behavior release system.^24^ We associate release of exosomes with the matrix degradation where after initial solubilization of a majority of the Q5 hydrogel, some exosomes remain within uneroded Q5 hydrogel matrix and are allowed to diffuse slowly between 2 and 72 h of the study. In comparison to the small molecule release studies of Q protein hydrogels previously, the lower stability of Q5Exo may be the result of lower structured content in Q5 after mixing with exosomes (Figure 1g). When mixed with curcumin, Q previously demonstrated an increase in helical and structured content, compared to Q alone^24^ indicating a stark difference in the impact of various encapsulated biomolecules on protein hydrogel secondary structure.

In comparison, there have been several notable examples of exosome-hydrogel hybrid biomaterials for accelerated diabetic wound healing. Included is the chitosan-grafted-dihydrocaffeic acid (CS-DA) and benzaldehyde-terminated Pluronic F127 (PF127-CHO) combined tannic acid (TA) hydrogels (CS-DA/PF/TA) for sustained release of 3D-cultured ADC-derived exosomes using C57 mice and a full-thickness 10 mm diameter skin wound model and single applications to the wound.^41^ CS-DA/PF/TA hydrogels have shown slight enhancement in would healing beyond exosome controls but have relied on site injection, which may provide increased retention. Other injectable hydrogels include hyaluronic acid (HA)-based hydrogels. These include MnO_2_/ε-PL nanosheet and fibril growth factor (FGF) scaffolded HA hydrogels (HA@MnO_2_/FGF-2) for encapsulation and delivery of exosomes, which revealed minor differences to the control vehicle group in C57 mice with full-thickness 1 cm^2^ skin wound models and injections at days 0, 3, 5, 7, and 9.^42^ Another example has been the F127/OHA-EPL (FHE) hydrogel, which releases exosomes from adipose-derived stem cells via pH-responsiveness, demonstrating slight improvements compared to exosomes alone with greatest differences seen at shorter times (7–14 days) in ICR mice with 8-mm-diameter full-thickness wounds using periodic applications to the wound at day 0, 3, 7, 14, and 21.^43^ In comparison, the FHE hydrogel alone shows substantially less improvement in the wound closure rate, while improvement of the hydrogel was still shown over that of the control group. Overall, the Q5Exo hydrogel demonstrates similar wound closure rates to exosomes alone, where slight improvements have been seen previously in synthetic-based hydrogels relying on injection application. Q5Exo and other hydrogel materials for the sustained release of exosomes benefit from improved bioavailability of exosomes during treatment. Thus, the tunability of hydrogel biomaterials may see additional benefits of improved release profiles. These hydrogel materials also provide improved hydration of the wound as a dressing, which has been reported to improve wound healing alone.^14^ Q5Exo may be considered an additional biomaterial among these vehicles for exosome delivery and subsequent wound healing, which benefits from topical application. Additionally, the completely protein-based design of Q5 allows for sequence modularity and tailoring of the material for improved exosome interaction, sustained release, and material strength of a coiled-coil protein and exosome hydrogel platform.

## Conclusions

We present a novel hybrid biomaterial system consisting of a tuned protein hydrogel capable of encapsulating exosomes. The resulting Q5Exo hydrogel allows for the release of exosomes for enhanced wound healing. In a Lepr^db/db^ diabetic mouse model, Q5Exo demonstrates similar wound closure time to exosomes alone and benefits from facile, topical application and does not require injection. Moreover, Q5 is detectable by its autofluorescence during administration, offering a window into monitoring the exosome delivery vehicle. Future work may allow for longer monitoring times atop a wound utilizing the inherent moiety of coiled-coil hydrogel systems. We demonstrate that encapsulation of exosomes by Q5 has a negligible effect on its material strength and strongly increases its rate of gelation by >2-fold in comparison to Q5 alone, allowing for additional ease in preparation and application of the Q5Exo system for wound healing. Furthermore, the impact of Q5Exo as a wound healing material offers important insight into the design considerations for hybrid protein-exosome delivery systems.

## Supporting Information Available

Representative 12% SDS-PAGE of protein purity after completion of biosynthesis steps, Q5Exo hydrogel on diabetic wound of mouse; sigmoidal fit analysis of microrheology assessed using MPT, replicates for microrheology and corresponding MPT superposition analysis, blood glucose measurements of diabetic mice in wound healing experiments (PDF)
