# Supplementary material for: Exosome Loaded Protein Hydrogel for Enhanced Gelation Kinetics and Wound Healing
Source: ACS Appl Bio Mater. 2024 Aug 22;7(9):5992–6000. doi: 10.1021/acsabm.4c00569 (PMC11409212; doi:10.1021/acsabm.4c00569)
Supplement: Supplementary file 1 — mt4c00569_si_001.pdf [file mt4c00569_si_001.pdf]

Supporting Information

Exosome Loaded Protein Hydrogel for Enhanced Gelation Kinetics and Wound Healing

Dustin Britton[†], *Dianny Almanzar*[§], *Yingxin Xiao*[†], *Hao-Wei Shih*[†], *Jakub Legocki*[†], *Piul Rabbani*[§], *Jin Kim Montclare*[†,¶,∇,°,¥,*]

† Department of Chemical and Biomolecular Engineering, New York University Tandon School of Engineering, Brooklyn, New York, 11201, USA

§ Hansjörg Wyss Department of Plastic Surgery, New York University School of Medicine, New York, New York, USA.

¶ Bernard and Irene Schwartz Center for Biomedical Imaging, Department of Radiology, New York University School of Medicine, New York, New York, 10016, USA

∇ Department of Chemistry, New York University, New York, New York, 10012, USA

° Department of Biomaterials, New York University College of Dentistry, New York, New York, 10010, USA

¥ Department of Biomedical Engineering, New York University, New York, NY, 11201, USA

* Corresponding author

Email: montclare@nyu.edu

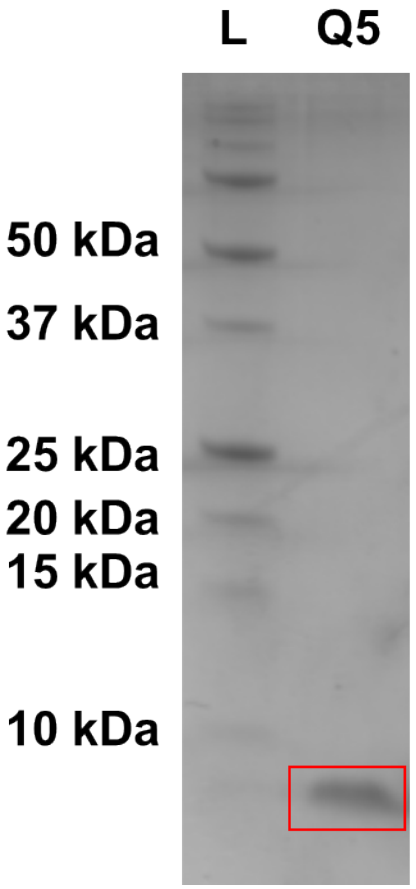

Figure S1 Representative 12% SDS-PAGE confirming purity of final Q5 protein (6.4 kDa) following expression, purification, dialysis, and concentration.

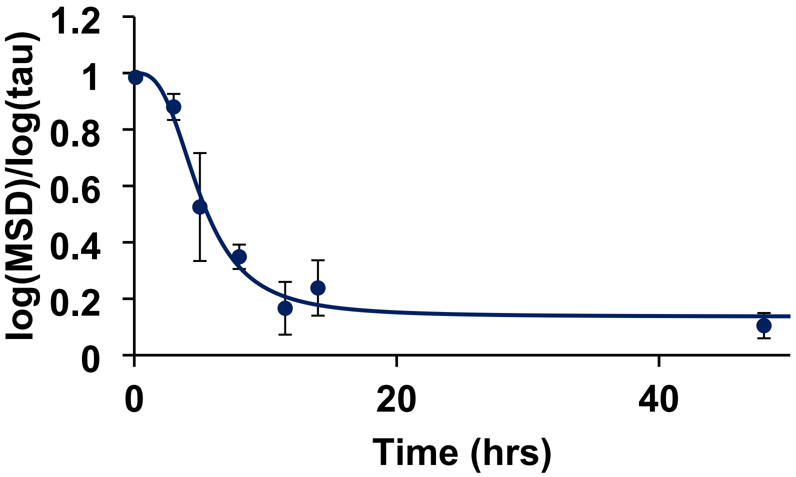

Figure S2 Sigmoidal fit of micrhreology curve using multiple particle tracking to assess logarithmic slope of the mean square displacement. Data is represented as the average and standard deviation of three independent trials.

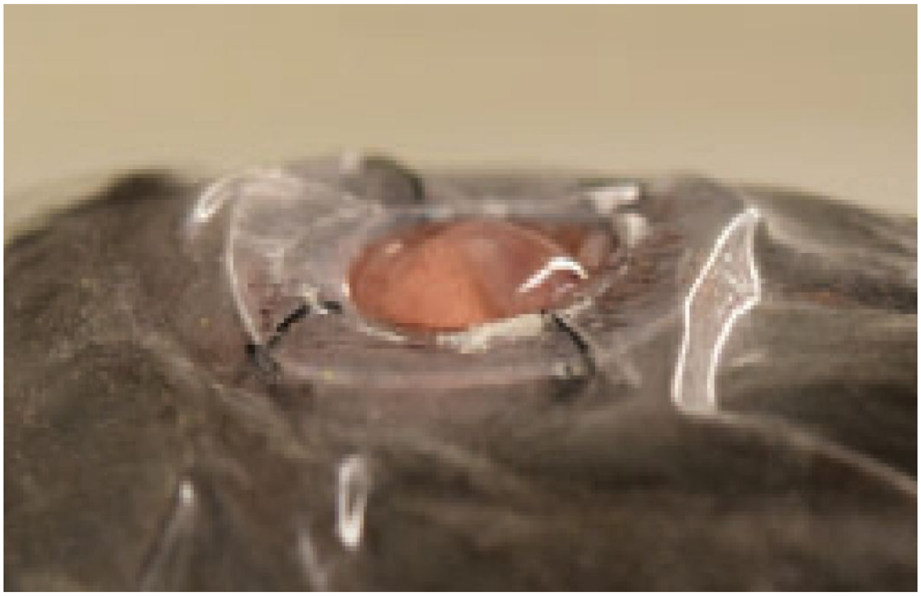

Figure S3 Photograph of wound on diabetic mouse immediately following application of Q5Exo hydrogel.

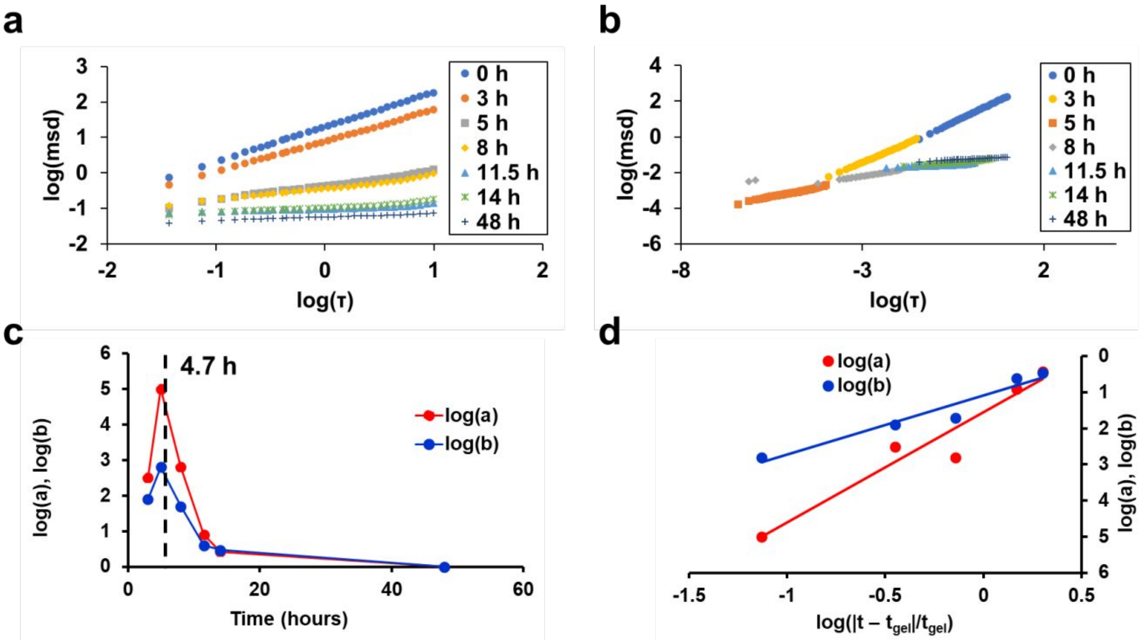

Figure S4 **a.** Log-log plot of MSD and lag time, τ, for independent trial (no. 2) of Q5Exo determined by MPT. **b.** Time-cure superposition of MSD vs. τ. **c.** Logarithmic shift factors for the vertical (log(a) in red) and horizontal (log(b) in blue) directions used in the time cure superposition to determine the t_{gel}. **d.** Log-log plot of the shift factors and their distance from t_{gel} determined by the ratio of the logarithmic slopes of the horizontal to vertical shift factor.

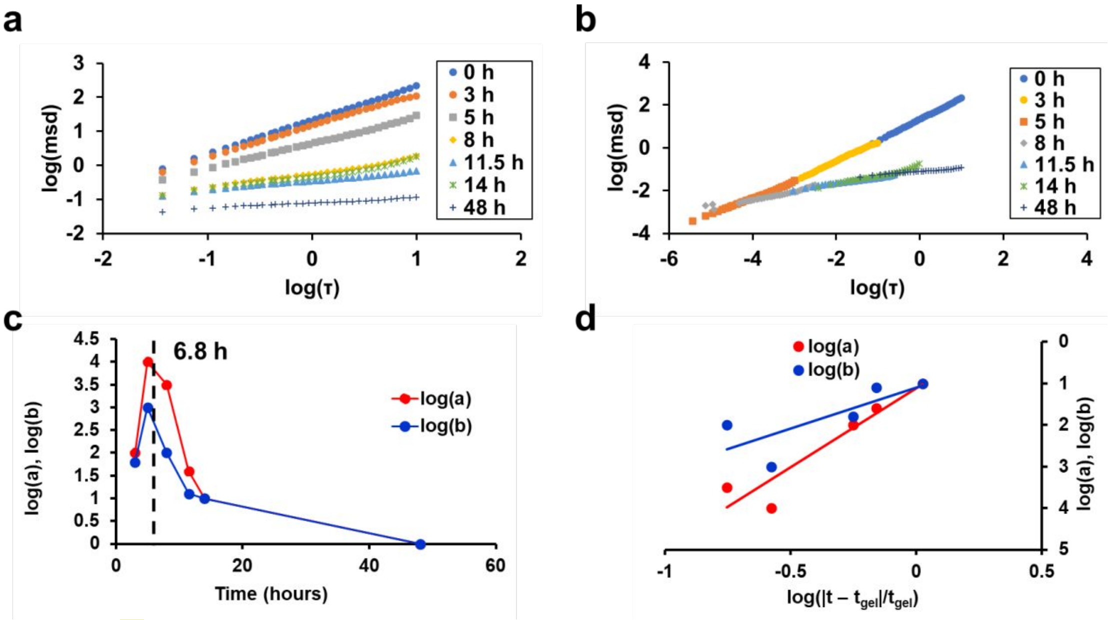

Figure S5 **a.** Log-log plot of MSD and lag time, τ, for independent trial (no. 3) of Q5Exo determined by MPT. **b.** Time-cure superposition of MSD vs. τ. **c.** Logarithmic shift factors for the vertical (log(a) in red) and horizontal (log(b) in blue) directions used in the time cure superposition to determine the t_{gel}. **d.** Log-log plot of the shift factors and their distance from t_{gel} determined by the ratio of the logarithmic slopes of the horizontal to vertical shift factor.

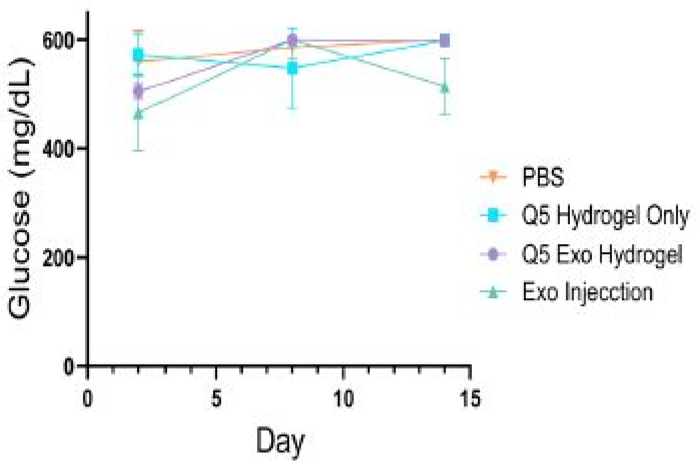

Figure S6 Blood glucose measurements in type 2 diabetic mouse model over course of wound monitoring. Glucose readings indicate that hyperglycemia ≥ 350 mg/dL, characteristic of diabetes, is not affected upon topical application of Q5Exo hydrogel or exosome injection. Data represents the average and standard deviation of ≥ 3 independent trials.
